# Effects of Physical Activity Level, Strength, Balance, and Body Composition on Perceived Health in Healthy Adults

**DOI:** 10.3390/sports13010019

**Published:** 2025-01-13

**Authors:** José Manuel Delfa-de-la-Morena, Pedro Pinheiro Paes, Frederico Camarotti Júnior, Débora Priscila Lima de Oliveira, Rubem Cordeiro Feitosa, Byanka Santos Cavalcante de Oliveira, Juan-José Mijarra-Murillo, Aranzazu Martínez Moya, Miriam García-González, Francisco De Asís-Fernández

**Affiliations:** 1 Department of Physical Therapy, Occupational Therapy, Rehabilitation and Physical Medicine, Rey Juan Carlos University, 28922 Madrid, Spain; jose.delfa@urjc.es (J.M.D.-d.-l.-M.); aranzazu.martinezm@urjc.es (A.M.M.); miriam.garciag@urjc.es (M.G.-G.); fasis.fernandez@urjc.es (F.D.A.-F.); 2 Cognitive Neuroscience, Pain, and Rehabilitation Research Group (NECODOR), Faculty of Health Sciences, Rey Juan Carlos University, 28922 Madrid, Spain; 3 Department of Physical Education, Federal University of Pernambuco, Recife 50670-901, PE, Brazil; pppaes@ufpe.br (P.P.P.); frederico.camarottijunior@ufpe.br (F.C.J.); debora.limaoliveira@ufpe.br (D.P.L.d.O.); rubem.cordeiro@ufpe.br (R.C.F.); byanka.cavalcante@ufpe.br (B.S.C.d.O.); 4 Research and Studies in Health and Performance Group (GEPPHS), Federal University of Pernambuco, Recife 50670-901, PE, Brazil; 5 International Doctoral School, Faculty of Health Sciences, Rey Juan Carlos University, 28922 Madrid, Spain

**Keywords:** adults, body composition, physical abilities, physical activity, quality of life

## Abstract

Background: Nowadays, not only is a high, long life expectancy desired, but also longevity with quality. Quality of life in adulthood is a multidimensional construct related to the perception of one’s own health, psychological and socio-emotional factors, functionality for daily activities, and body composition. Objective: This study evaluates the effects of physical activity level (PAL), strength, balance, and body composition on perceived health in healthy adults. Methods: An observational, cross-sectional study with consecutive, non-probabilistic inclusion of cases was conducted. Body fat percentage (BFP) was measured by DXA. Physical activity level was assessed using accelerometry. The strength index (S_Index) was estimated using dynamometry. Postural control was assessed through posturography. The composite equilibrium score from the Sensory Organization Test (SOT_CES) was conducted to measure postural stability under various sensory conditions using dynamic posturography. Perceived health was calculated using the SF36 questionnaire, which detects health states, both positive and negative. A linear regression model was generated between each domain of SF36 with SOT_CES, BFP, PAL, and S_Index. Results: A total of 64 males with a mean age of 55 ± 5 years and a mean body mass index of 27 ± 4 kg/m^2^ were recruited. Results showed a negative correlation between physical function (ß = −0.7; t = −3.163; *p* = 0.003; R^2^ = 23.7%) and general health (ß = −0.227; t = −3.425; *p* = 0.001; R^2^ = 17.4%) with BFP. Also, it showed a negative correlation between physical function (ß = 0.047; t = −2.643; *p* = 0.011; R^2^ = 17.5%) and general health (ß = 0.016; t = −3.044; *p* = 0.004; R^2^ = 14.6%) with S_Index. On the other hand, no relation was observed between SF36 and SOT_CES. Finally, only the emotional role showed a positive correlation (ß = −0.02; t = −2.629; *p* = 0.011; R^2^ = 23.1%) with PAL. Conclusion: A lower BFP and higher S_Index are associated with increased physical function and general health. Also, the higher the PAL, the greater the emotional health. On the other hand, no relation was observed between SF36 and the balance detected from SOT_CES.

## 1. Introduction

Quality of life in adulthood and the elderly is a multidimensional construct that is related to various factors, such as perception of one’s own health and comparison with one’s peers, psychological and socio-emotional factors, physical skills (e.g., strength, balance, flexibility), independence and functionality for daily activities, and body composition (in terms of fat percentage and muscle mass) [1]. Nowadays, not only is a high, long life expectancy desired, but also longevity with quality of life [2].

The transition from adulthood to old age is marked by psychophysiological changes that impact and are impacted by the variables mentioned above [3,4]. In this scenario, physical activity plays a fundamental role in modulating this interrelationship, being associated with quality of life in a directly proportional way and positively influencing the body’s physical and mental capacities [3]. According to the study by Strain et al. [5] of 5.7 million adults around the world between 2000 and 2022, the prevalence of insufficient levels of physical activity has increased in 103 of the 197 countries and in 6 of the 9 regions assessed globally.

High levels of physical inactivity in this age group are related to loss of muscle mass (sarcopenia) and an increase in weight and body fat percentage, which are serious risk factors for the onset of various chronic non-communicable diseases and negatively affect cardiovascular and metabolic health [6]. Research has shown that the loss of muscle mass accompanied by an increase in adiposity (body fat) is associated with a decrease in quality of life and an increased risk of mortality [6].

According to Ekelund et al. [7], being physically active improves the perception of health and quality of life in all age groups, reduces body fat, increases muscle mass, and improves motor skills such as strength and balance. Strength is a vital physical capacity that is directly related to a better perception of health; better levels of strength contribute to adequate body composition (more muscle, less fat, improvements in bone mineral density, and postural stability [8]). Several researchers attest that muscle strength is a relevant aspect of well-being, directly and positively associated with quality of life, while sarcopenia is associated with a lower perception of health [9,10,11].

In the same vein, balance, the physical ability to maintain an upright posture in opposition to gravity and other internal and external forces on the body, is essential for maintaining a good quality of life since, with advancing age, this ability is weakened (along with loss of muscle mass), and this combination becomes an important risk factor for the increase in falls in older adults and especially the elderly, often leaving sequelae, such as loss of independence for daily activities, negatively impacting quality of life and mental well-being [10,12,13].

In addition to the physical aspects, an active lifestyle, whether through everyday physical activities, exercise, or sports, can generate psycho-emotional benefits in adults and the elderly through interaction and socialization practices, combating some disorders such as anxiety and depression, and strengthening physiological functions and musculoskeletal structures, which confer greater functionality [14]. This contributes to a positive perception of general health and mental well-being, resulting in a good quality of life.

There are few analyses that focus on understanding how these variables interact; many do so in isolation or in pairs. This leaves a gap to be filled, which would be to demonstrate how the various dimensions of human quality of life communicate with each other. Thus, the aim of this study was to evaluate the effects of physical activity level, strength, balance, and body composition on perceived health in healthy adults.

## 2. Materials and Methods

### 2.1. Study Design

This study is observational and cross-sectional, with consecutive non-probabilistic case inclusion. All participants received written information about the nature and purpose of the study and provided their informed consent before the research began. The study protocol, in line with the Declaration of Helsinki on research involving human subjects, was approved by the Ethics Committee of Rey Juan Carlos University (number: 300120170241). The trial is registered on ClinicalTrials.gov with the ID: NCT01116856 (http://clinicaltrials.gov/study/NCT01116856, accessed on 5 May 2010).

### 2.2. Participants

This study included healthy males aged 18 and over. Individuals with serious illnesses, smokers or those who had recently quit smoking (within the past six months), those who consumed alcohol, had a history of balance disorders, had undergone knee or hip replacement surgery, or experienced lower limb trauma in the last six months, or had arthritis or other severe inflammatory diseases in the lower limbs were excluded.

An email was sent to participants in the PRONAF study (Programme of Nutrition and Physical Activity to Control Obesity) seeking volunteers to take part in this study [15]. A total of 131 people showed interest in taking part in the research. Of these, only 67 satisfied the inclusion criteria. During the data collection period, three participants dropped out for personal reasons, so the final sample was 64 people.

### 2.3. Outcomes

Anthropometric data included measurements of weight and height. Weight was assessed using a Tanita^®^ BC-420MA scale (Bio Lógica Tecnología Médica S.L, Barcelona, Spain), while height was measured using a Seca^®^ stadiometer (range: 80–200 cm), manufactured by Seca GmbH & Co. KG (Hamburg, Germany). These values were then used to calculate the body mass index (BMI). Participants were classified as obese if their BMI was 30 kg/m^2^ or higher, overweight if their BMI was between 25 and 29.9 kg/m^2^, and normal weight if their BMI was below 25 kg/m^2^ [16].

All participants were given the Spanish-validated version of the International Questionnaire of Physical Activity (IPAQ), which assessed their physical activity.

Body composition variables were measured by dual X-ray absorptiometry (DXA) [17] using a GE Lunar Prodigy densitometer (GE Healthcare, Madison, WI, USA). Only body fat percentages from all body composition parameters were registered for this study.

Physical activity was evaluated using the SenseWear^®^ Armband (SWA), an objective, multisensory device known for its validity and reliability in measuring physical activity [18,19]. The Physical Activity Level (PAL) test [20] was utilized to categorize participants as either sedentary or active. Those with a PAL classification of “not very active”, “active”, or “very active” (PAL ≥ 1.4) were grouped into the “Physical Activity Group”, while participants with a PAL value below 1.4 were classified as the “Sedentary Group”.

The strength index S_Index was calculated by using a Tecsymp Tkk5002 hand dynamometer and a Tecsymp Tkk5401 leg and back dynamometer (Tecsymp, Barcelona, Spain) for measuring muscular strength. The S_Index value was determined by summing the values obtained from the three instruments and dividing by the subject’s body weight.

The composite equilibrium score from the Sensory Organization Test (SOT_CES) was conducted to measure postural stability under various sensory conditions using dynamic posturography (Equi Test: Neurocom International, Clackamas, OR, USA). The Equi Test system features a force platform and a visual environment that can either stay fixed or become mobile, rotating around the ankle joints in response to the individual’s postural adjustments. This system, used with either open or closed eyes, provides insights into the somatosensory, visual, and vestibular contributions to balance. The test involved six conditions: (a) open eyes, fixed visual environment, and fixed support; (b) closed eyes, fixed support; (c) mobile visual environment and fixed support; (d) fixed visual environment and mobile support; (e) closed eyes, mobile support; and (f) open eyes, mobile visual environment, and mobile support. Each condition was measured three times for 20 s to obtain an average value [21].

The health perceived SF36 questionnaire was distributed to all participants. Test SF36 is a questionnaire on quality of life consisting of 36 questions (items), which detect health states, both positive and negative [22]. The questionnaire is concluded with two summary components (physical and mental) from 8 dimensions: physical function (10 items), physical role (4 items), emotional role (3 items), social function (2 items), health mental (5 items), general health (5 items), bodily pain (2 items) and vitality (4 items). It also contains an additional item that is not part of any dimension and measures the change in health over time (1 item).

### 2.4. Statistical Analysis

Descriptive statistics for continuous variables are summarized as mean ± standard deviation. A linear regression model was generated using Ordinary Least Squares (OLS) between each dimension (physical function, physical role, emotional role, social function, mental health, general health, bodily pain, and vitality) of SF36 with SOT_CES, body fat percentage (BFP), PAL, and strength index (SI) [23]. To evaluate if the regression model is adequate, the model assumptions were tested (linearity, homoscedasticity of the residuals, and normality of the residuals). Scatter plots and Q-Q plots were generated to visually analyze the model. Once the model was assumed, the unstandardized coefficient, the 95% confidence interval, t-student, and the *p*-value were reported.

All analyses were performed with the Statistical Package for the Social Sciences (SPSS) 23.0, with a confidence of 95%, considering statistical significance when *p* < 0.05.

## 3. Results

The sample consisted of 64 males with a mean age of 55 ± 5 (41 to 67) years and a mean body mass index (BMI) of 27 ± 4 kg/m^2^; 43.75% were classified as obese, 43.75% were classified as overweight, and 12.5% as normal weight. The daily physical activity of participants was 2.64 ± 1.21, and the weekly METs were 3604 ± 3048 for the IPAQ. A total of 61% were classified as physically active, and 39% were classified as sedentary.

All descriptive information is presented in Table 1.

### 3.1. Body Fat Percentage and Perceived Health

Once the model was assumed between each dimension of SF36 and BFP (SuppData S1), the unstandardized coefficient, t-value, r-value, *p*-value, and 95% confidence interval were reported in Table 2. Results showed a negative correlation between physical function (ß = −0.7; t = −3.163; *p* = 0.003; R^2^ = 23.7%) and general health (ß = −0.227; t = −3.425; *p* = 0.001; R^2^ = 17.4%) with BFP; on the other hand, it showed a positive correlation between physical role (ß = 0.204; t = −2.799; *p* = 0.007; R^2^ = 20.1%) with BFP.

### 3.2. Physical Activity Level and Perceived Health

After the model was applied to each dimension of SF36 and PAL (SuppData S2), the unstandardized coefficient, t-value, r-value, *p*-value, and the 95% confidence interval were reported in Table 3. Results showed no correlation between the perceived health dimensions reflected by the SF36 test with PAL except with the emotional role (ß = 0.20; t = −2.629; *p* = 0.011; R^2^ = 23.1%).

### 3.3. Sensorial Organization Test and Perceived Health

Once the model was established for each dimension of SF36 and SOT-CES (SuppData S3), the unstandardized coefficient, t-value, r-value, *p*-value, and 95% confidence interval were reported in Table 4. Results showed no correlation between the perceived health dimensions reflected by the SF36 test and the posturography reflected in the SOT-CES.

### 3.4. Strength Index and Perceived Health

When the model was assumed across the various dimensions of SF36 and S_INDEX (SuppData S4), the unstandardized coefficient, r-value, t-value, *p*-value, and the 95% confidence interval were reported in Table 5. Results showed a positive correlation between physical function (ß = 0.047; t = −2.643; *p* = 0.011; R^2^ = 17.5%) and general health (ß = 0.016; t = −3.044; *p* = 0.004; R^2^ = 14.6%) with S_INDEX; on the other hand, it showed a negative correlation between physical role (ß = −0.015; t = −2.498; *p* = 0.016; R^2^ = 17%) with S_INDEX.

## 4. Discussion

This investigation sought to verify the relationship between quality of life, level of physical activity, strength, balance, and body composition in adults. This justifies the relevance of these variables, which have been studied, albeit separately, for the promotion of healthy aging, which is known to be interdependent and multifactorial. This reinforces the importance of strategies and studies with a holistic health perspective. De Maio Nascimento et al. [24] highlight the importance of studies of this nature in their research, which showed that these variables play a significant role in quality of life.

Sok et al. [25], in a multicomponent integrative health intervention with older adults, showed improvements in quality of life, overall health status, and cognitive function. The findings reinforce that interventions focused on strength and balance can be fundamental strategies for promoting healthy aging and greater functional autonomy. Reflecting on a quality of life that is based on fundamental pillars, such as a good level of physical activity and consequently functional physical capacities (e.g., muscle strength and balance), body composition appropriate for weight and age, and a good psycho-emotional state.

The total sample consisted of 64 individuals, aged between 41 and 67 years (mean age 55 ± 5), and based on the Metabolic Equivalents of Task—METs (average between 3 and 6 METs), the sample was considered physically active [26]. This runs counter to global trends of greater physical inactivity and insufficient levels of body movement, as shown in a worldwide study carried out on all continents, which showed this increase associated with advancing age [5]. This raises questions about the importance of being physically active and the multidimensionality of health and quality of life. In this scenario, promoting physical activity is an interesting strategy [27].

Statistical correlations showed a significant value (Beta = 0.481) between the level of physical activity and emotional role (SF-36). This shows that the more physical activity one does, the greater the psycho-emotional benefits generated by the socialization inherent in these practices.

The research by De Maio Nascimento et al. [24] found that physical activity is positively related to mental health, with balance and muscle strengthening modulating its impact on quality of life, especially in the mental sphere. On the other hand, resistance training not only improved physical abilities but also reduced symptoms related to the perception of frailty, which contributed to better mental and emotional health in the elderly due to the social interaction that the moment of practice offers, often creating friendships and a sense of belonging to a group [27].

However, depending on the amount and intensity of physical activity or environments that are not very welcoming, the effect of physical activity on mental well-being can be reduced or even reversed, as shown in the study by Nakagawa et al. [28]. Furthermore, in addition to mental aspects, physical activity is also positively associated with strength, the risk of falls, balance, and body composition [29].

A significant and negative correlation was found between the percentage of body fat and physical function (Beta = −0.487) and general health (Beta = 0.418), where each point that the percentage of body fat decreases increases the repertoire of physical abilities by 0.7. In other words, the higher levels of physical activity of the sample of participants in this study were accompanied by lower percentages of body fat, [30] agree by arguing that the percentage of fat is inversely proportional to the amount of muscle mass, and if the percentage is excessive or inadequate, it causes damage to individuals’ perception of their quality of life. Because they are related to chronic diseases, they have less autonomy and postural control.

Physical activity, together with other healthy lifestyle habits, can improve physical functionality and overall well-being by combating overweight (changes in body composition mostly due to increased fat) and sarcopenia (progressive loss of muscle over the years) [31]. While body composition can influence balance, a greater lean mass represents more muscle and more strength to counterbalance imbalances and prevent falls; a higher level of fat mass can alter the body’s center of gravity, generating greater postural instability [32].

Regarding balance, as assessed by the Sensory Organization Test (SOT), there was no correlation between the posturography data and the physical or mental health and quality of life scores on the SF-36. However, the percentage of fat can generate some alterations in the physical aspects of balance; the leaner mass they had, the better their balance levels [20].

This ability is multidimensional and depends on the interaction, mainly of the visual, vestibular, and proprioceptive systems (in the muscles and joints), and has reverberations with body composition, levels of physical activity, strength, and quality of life [33,34]. Jeong et al. [20] argue that a higher percentage of fat and body mass can compromise functionality and balance by altering the body’s center of gravity and gait pattern and is associated with lower lean mass (muscles–strength).

In the study by Onofrei and Amaricai [35], it was postulated that higher levels of physical activity are a fundamental indicator of the relationship between postural control and body composition. Furthermore, with a view to a good quality of life over the years, Adams et al. [36] argue that greater postural stability and strength levels help to cope with the decline in body functionality linked to advancing age. This disagrees with the findings of the present study, which found no correlation between quality of life and balance and strength.

Similarly to balance, the physical valence of strength also showed no correlation with the perception of health and quality of life of the volunteers in this study. However, in a negative correlation, it was found that the lower the percentage of body fat, the higher the level of strength and muscle. In addition, strength showed a positive correlation with general health and physical function scores but without statistical significance. This agrees with some studies that have shown a positive association between greater PAL and increased strength, generating improvements in body composition by increasing mass and reducing fat [37,38].

In this sense, body composition has a direct impact on balance, and individuals with lean mass maintain better strength and balance, factors that promote a better perception of health and well-being [39]. This is in line with the inverse relationship found in this study between fat percentage and muscle strength and the correlation between strength and general health score. Halaweh [40], when assessing handgrip strength in adults, indicated that maintaining a good strength score can help improve health-related quality of life. Increasing the ability to perform everyday tasks impacts good self-esteem and mental health through improved, maintained physical function.

This study has certain limitations, including the lack of a control group to provide a baseline for comparisons. As this is a cross-sectional study, care should be taken when drawing strong conclusions about causality. The sample was made up of male adults from Spain, so the results cannot be generalized to regions and countries with very different environmental and socio-cultural characteristics. Other limitations may be the small sample and the age discrepancy. Also, the classification of obesity and overweight was performed on the basis of BMI when a DEXA was available. It might be that a classification based on body fat percentage would have been more appropriate.

It is worth noting that there are still few studies in the literature with this target audience that assess the relationship between these variables in an interconnected way, and it is recommended that more research be carried out longitudinally to provide more robust and comprehensive evidence. Future studies should consider other physical capacities, such as cardiorespiratory ones, and larger samples with different anthropometric, socioeconomic, and regional profiles to broaden and strengthen new inferences about these variables in this public.

The findings of this research contribute to building a theoretical framework that supports the planning and execution of further studies and interventions across various fields, including physical education, nutrition, physiotherapy, and medicine. These results may have practical implications for the implementation of both public and private health programs aimed at enhancing the overall quality of life for adults and the elderly. Specifically, they underscore the importance of conducting regular assessments of physical abilities and psycho-emotional states. At this stage of life, adopting a healthy lifestyle and habits is important, as they promote both physical and mental well-being, ensuring sustained quality of life throughout the aging process.

## 5. Conclusions

It can be concluded that a lower BFP and higher S_Index increased physical function and general health. Also, the higher the PAL, the higher the emotional role. On the other hand, no relation was observed between SF36 and the balance detected from SOT_CES.

## Figures and Tables

**Table 1:** Descriptive data of participants (n = 64).

	Min	Max	Mean	SD
AGE (years)	41	67	55.17	5.71
BMI	20.73	49.01	27.86	4.55
BFP (%)	10.41	44.22	27.40	8.70
IPAQ (METs)	0	17346	3604	3048
PAL	0.00	5.90	2.64	1.21
SOT_CES	60	89	77.86	6.12
S_INDEX	2.43	5.81	4.16	0.69
Physical function (0–100)	65	100	97.11	6.09
Physical role (0–100)	0	100	94.14	19.28
Bodily pain (0–100)	22	100	80.69	18.62
General health (0–100)	45	100	74.16	16.15
Vitality (0–100)	20	100	70.62	16.39
Social function (0–100)	13	100	90.82	16.25
Emotional role (0–100)	0	100	89.58	29.02
Mental health (0–100)	28	100	79.87	13.31

BMI, body mass index; BFP, body fat percentage; PAL, physical activity level; SOT_CES, composite equilibrium score from sensory organization test; S_INDEX, strength index.

**Table 2:** Summarized table of linear regression between predicted variable (SF36) and predictor variable (BFP body fat percentage).

	Unstandardized Coefficient		R^2^	t	*p*-Value	95% CI for B	
	B	Std. Error					
Physical function	−0.700	0.221	0.237	−30.163	0.003	−10.144	−0.256
Physical role	0.204	0.073	0.201	20.799	0.007	0.058	0.350
Bodily pain	0.002	0.065	0.000	0.031	0.976	−0.128	0.132
General health	−0.227	0.066	0.174	−30.425	0.001	−0.360	−0.094
Vitality	0.062	0.088	0.013	0.704	0.485	−0.114	0.238
Social function	−0.066	0.116	0.015	−0.573	0.569	−0.298	0.165
Emotional role	−0.025	0.050	0.006	−0.499	0.620	−0.126	0.076
Mental health	0.099	0.124	0.022	0.795	0.430	−0.150	0.348

**Table 3:** Summarized table of linear regression between predicted variable (SF36) and predictor variable (PAL physical activity level).

	Unstandardized Coefficient		R^2^	t	*p*-Value	95% CI for B	
	B	Std. Error					
Physical function	0.042	0.034	0.044	10.242	0.220	−0.026	0.109
Physical role	−0.001	0.011	0.000	−0.124	0.902	−0.023	0.021
Bodily pain	−0.004	0.010	0.004	−0.425	0.673	−0.024	0.015
General health	0.013	0.010	0.029	10.275	0.208	−0.007	0.033
Vitality	0.019	0.013	0.063	10.397	0.168	−0.008	0.045
Social function	−0.014	0.017	0.034	−0.783	0.437	−0.049	0.021
Emotional role	0.020	0.008	0.231	20.629	0.011	0.005	0.035
Mental health	−0.033	0.019	0.128	−10.732	0.089	−0.070	0.005

**Table 4:** Summarized table of linear regression between predicted variable (SF36) and predictor variable (SOT-CES composite equilibrium score from sensory organization test).

	Unstandardized Coefficient		R^2^	t	*p*-Value	95% CI for B	
	B	Std. Error					
Physical function	0.087	0.182	0.008	0.479	0.634	−0.277	0.451
Physical role	−0.056	0.060	0.031	−0.940	0.351	−0.176	0.064
Bodily pain	0.041	0.053	0.015	0.769	0.445	−0.066	0.147
General health	0.025	0.054	0.004	0.451	0.654	−0.084	0.133
Vitality	0.055	0.072	0.022	0.767	0.446	−0.089	0.200
Social function	0.143	0.095	0.144	10.505	0.138	−0.047	0.333
Emotional role	−0.057	0.041	0.073	−10.380	0.173	−0.140	0.026
Mental health	−0.092	0.102	0.040	−0.900	0.372	−0.296	0.113

**Table 5:** Summarized table of linear regression between predicted variable (SF36) and predictor variable (S_INDEX strength index).

	Unstandardized Coefficient		R^2^	t	*p*-Value	95% CI for B	
	B	Std. Error					
Physical function	0.047	0.018	0.175	20.643	0.011	0.011	0.083
Physical role	−0.015	0.006	0.170	−20.498	0.016	−0.027	−0.003
Bodily pain	0.002	0.005	0.004	0.445	0.658	−0.008	0.013
General health	0.016	0.005	0.146	30.044	0.004	0.006	0.027
Vitality	−0.004	0.007	0.009	−0.564	0.575	−0.018	0.010
Social function	0.010	0.009	0.060	10.107	0.273	−0.008	0.029
Emotional role	−0.006	0.004	0.068	−10.522	0.134	−0.014	0.002
Mental health	0.001	0.010	0.001	0.141	0.888	−0.019	0.022

## Data Availability

The data presented in this study are available on request from the first author. Data are unavailable due to privacy or ethical restrictions.

## Supplementary Materials

The following supporting information can be downloaded at: https://www.mdpi.com/article/10.3390/sports13010019/s1, SuppData S1: Model Assumption BFP and SF36; SuppData S2: Model Assumption PAL and SF36; SuppData S3: Model Assumption SOT and SF36; SuppData S4: Model Assumption S_INDEX and SF36.
