# Supplementary material for: Effects of Physical Activity Level, Strength, Balance, and Body Composition on Perceived Health in Healthy Adults
Source: Sports (Basel). 2025 Jan 13;13(1):19. doi: 10.3390/sports13010019 (PMC11768831; doi:10.3390/sports13010019)

Supplementary Data3. Model Assumption SOT-CES and SF36

1º. Histogram (x=SOT-CES y =SF36)

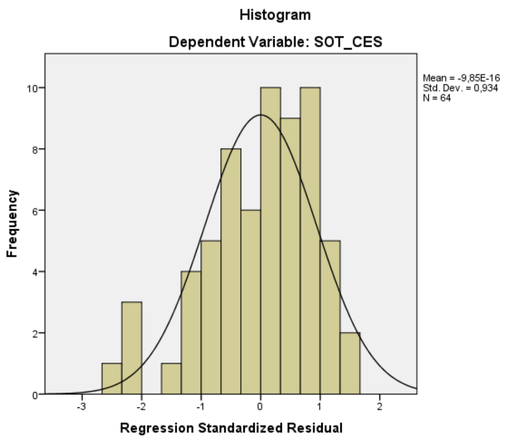

2º. QQ plot

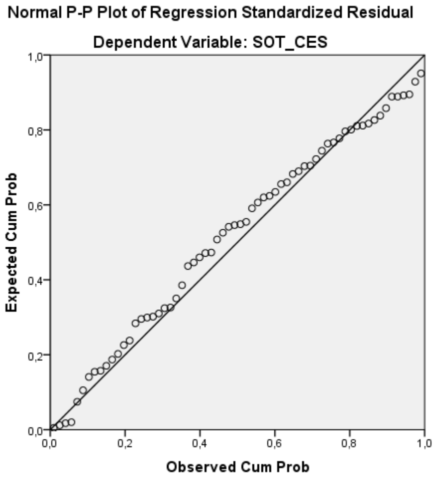

3º Homoscedasticity (x=ZPRED y =ZRESID)

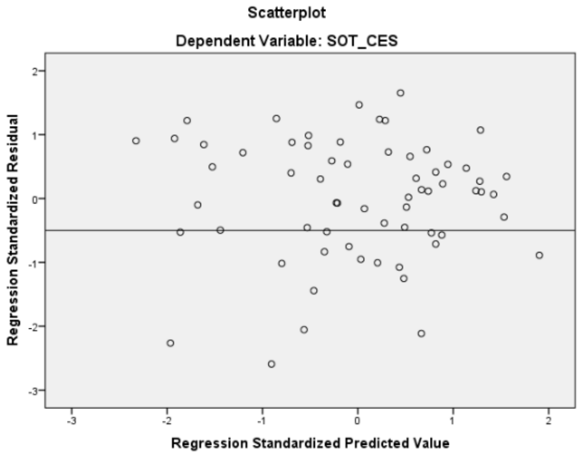

Supplement: Supplementary file 1 [file sports-13-00019-s001.zip › SuppData S3. Model Assumption SOT and SF36.pdf]
